# Phytoremediation of benzene, toluene, ethylbenzene and xylene contaminated air by *D. deremensis* and *O. microdasys* plants

**DOI:** 10.1186/2052-336X-12-39

**Published:** 2014-01-22

**Authors:** Mohammad Hossein Mosaddegh, Abbas Jafarian, Adele Ghasemi, Alimohammad Mosaddegh

**Affiliations:** 1 Department of Pharmacology and Toxicology, School of Pharmacy and Pharmaceutical Sciences, Yazd Shahid Sadoughi University of Medical Sciences, Yazd, Iran; 2 Department of Pharmacolgy and Toxicology, School of Pharmacy and Pharmaceutical Sciences, Isfahan University of Medical Sciences, Isfahan, Iran; 3 School of Pharmacy and Pharmaceutical Sciences, Yazd Shahid Sadoughi University of Medical Sciences, Yazd, Iran

**Keywords:** BTEX, D. deremensis, O. microdasys

## Abstract

**Background:**

People usually spent about 90% of their time indoors, which are probably more polluted than outside the buildings. High levels of volatile organic compounds (VOCs) are known as causes of sick building syndrome. The present study was designed to determine the quantitative effects of some plants to improve the quality of the environmental air.

**Results:**

*D. deremensis* and *O. microdasys* were chosen for the present study. There is no report of using *O. microdasys* for cleaning the air from pollutants. So, in this study, the effectiveness of *O. microdasys* in air removing from pollutants was studied and compared with *D. dermensis*.

*O. microdasys* plant can remove 2 ppm concentration benzene, toluene, xylene and ethylbenzene from air in test chambers completely after 48, 55, 47 and 57 hours, respectively. The removal rates of benzene, toluene, xylene and ethylbenzene (BTEX) from air in the test chambers were 1.18, 0.54, 1.64 and 1.35 mg/ m^2^d^1^, respectively.

**Conclusions:**

If an office containing 2.5 ppm of each of BTEX and had an approximate volume of 30 m^3^, it contains 16, 8, 22 and 22 mg/m^3^ benzene, toluene, xylene and ethylbenzene, respectively. Using ten O. microdasys pots with the same size used in this study, can remove benzene, toluene, xylene and ethylbenzene totally after 36, 40, 30 and 39 hours.

The authors recommended studying the efficiency of the plants for removal of BTEX from air at higher range of concentrations such as 20-30 ppm.

## Background

People generally spend about 90% of their time inside the buildings such as houses, offices and factories (indoor). The indoor air is probably more polluted than that of the outside buildings air (outdoor). Therefore, the possible effects of air pollution on human beings are an international issue [1-4]. Volatile organic compounds (VOCs) are the most important pollutants of indoor air [4,5]. Average indoor levels of VOCs may be several times more than that of the outdoor air [6-8]. High levels of VOCs are known as causes of building related illness or sick building syndrome [5,9,10]. Several studies have reported the effects of some plants on the improvement of indoor air quality by absorbing air-borne contaminants such as VOCs [11-14].

Staff welfare and productivity were improved by putting plants indoor [15,16]. Several indoor plant species have eliminated benzene or hexane at levels of 50 and 150 ppm, respectively [17-19]. These concentrations are several orders of magnitude more than the levels that can be encountered in the indoor air.

It has been showed that one of the VOCs removal agents are microorganisms of the soil. But, there was not significant effect on the VOCs removal for the majority of air contaminants [18,19]. Also, it was proven that soil microorganisms can humiliate petroleum hydrocarbons in liquid phase form. Studies such as those conducted by Leigh et al., Margesin et al., and Chaianeau et al. forms the basis of bioremediation [20-22].

Godish and Guindon reported the effects of spider plants on the removal of formaldehyde up to 50%. They showed that the removal effects of the plants were not primarily via the plants leaves. As they concluded that it must be rather due to other factors such as moisture, soil, microorganisms or all of them [23]. Schmitz et al. studied the effects of 27 indoor plants on the activity of formaldehyde dehydrogenase and formate dehyrogenase. They reported that there was no significant effect on formaldehyde metabolism or uptake from stomata of the plants [24].

Burchett et al. examined the capacity of three plants named *Aglaonema modestum, Chamaedoreae legans* and *Philodendron* for cleaning the environment from benzene, toluene, xylene and n-hexane, which are used as industrial solvents for furnishings. They showed that the contaminants concentrations were decreased gradually to below the detection limits of the gas chromatograph (<20 ppb). They found that the pot size in cleaning of the air is in less importance [25].

Reduction of VOCs levels especially in indoor environments is vital to improve human health. So, the present study was designed to determine the quantitative effects of *Dracaena deremensis* and *Opuntia microdasy* to improve the quality of the environmental air.

## Methods

### Chemicals and supplies

Benzene, toluene, ethylbenzene, xylene, methanol, acetone and acetonitrile (HPLC grade) were purchased from Merck. All SPME supplies were obtained from Supelco. A CAR/PDMS fiber (75 μm) was used for sampling benzene from air. For sampling, EconoGrab 10 L Tedlar Bag with polyproplyene fitting and gas sampling pump (obtained from SKC, UK) were used. The Tedlar bags after each sampling were purged three times with nitrogen for reuse.

### Selected plants

*D. deremensis* and *O. microdasys* were chosen for the present study. The capability of *D. dermensis* for removing of air pollutants such as benzene and toluene was confirmed by Orwell et al (26). There is no report of using *O. microdasys* for cleaning the air from pollutants. So, in this study, the effectiveness of *O. microdasys* in comparison with *D. dermensis* in air removing from pollutants was studied. Three year old plants were transferred to 10 cm diameter pots. Then, the pots left for two months in the laboratory to get habit with the new conditions. The temperature was kept at 20°C ± 3 and the light period was 12/12 hours dark/light. The plants were watered each three days and the remaining water was allowed to drain from the pots. All plants were watered one hour before putting under exposure to gas mixture. The characteristic of the plants are listed in the Table 1.

**Table 1:** Features of used plants and pots

**Species**	**Plant age (year)**	**Leaf area (cm**^**2**^**)**	**Pot diameter (cm)**
D. deremensis	3	1380 cm^2^	10
O. microdasys	3	1350 cm^2^	10

The plants were placed in the sealed chambers. After an hour, gas treatment was imposed on the plants. The plants with about the same leaf areas were placed in the chambers. Three replicates of each plant were examined for the gas treatment. The empty chambers without plants were tested as control chambers. It was used to determine the losses caused by leakage, chemical reaction or adsorption to the chamber surface. Leaves area of the plants were measured at the end of the test. At the end of each experiment, the plant was extracted from the soil and the pot was placed in the chamber to examine the gas absorption capacity of the soil alone.

### Apparatus

Glass chambers with the internal volume of 0.05 m^3^ were purchased. Their diameter mouths were 35 cm that ease putting the pots inside the chamber. Removable glass lids were prepared and sealed with a solvent free adhesive tape. Rubber septa were installed on the centre of the lids for benzene, toluene, ethylbenzene and xylene injection and air sampling. A 2.4 W fan, controlled with remote, was installed inside the chambers to accelerate atmospheric equilibration. After benzene, toluene, ethylbenzene and xylene loading in the chamber, the fan was turned on for one hour, which is necessary for equilibration. As well, one hour before each sampling the fan was turned on to equilibrate benzene, toluene, ethylbenzene and xylene inside the chamber. Solid phase microextraction (SPME) fiber, CAR/PDMS fiber (75 μm), was used for their extraction from the chamber. The fiber was put inside the chamber in contact with the air and left it for equilibration. Then, the fiber was extracted from the chamber and inserted into the injection port for desorption.

Gas chromatography (Younglin series YL 6100) with flame ionization detector was used to determine benzene concentration in the air. A capillary column DB-5MS (J & W Scientific) fused silica (60 m, 0.25 mm i.d., 0.25 μm film thickness) was used. The operating conditions were: hydrogen flow 30 ml/min., air flow 300 ml/min, helium flow 0.8 ml/min, injector and detector temperatures were 260, 280°C, respectively. The GC oven was held at 40°C for 1 min, it was then ramped at 15°C/min to 90°C and held for 4 min, and finally ramped at 10°C/min to 170°C/min and held for 4 min. For calibrations, appropriate amounts of benzene, toluene, ethylbenzene and xylene and methanol (internal standard) were put into a 10 L Tedlar bag and after equilibration they were extracted using a CAR/PDMS fiber (75 μm film thickness). Standard curves were consistently linear with R2 > 0.98. Quantitation was based on peak area ratio, which is relation between their concentrations to methanol concentration.

### Procedure

The plants were watered and left for one hour to drain the extra water. Then, the plants were placed one in each chamber and the lids were sealed by adhesive. Benzene, toluene, ethylbenzene and xylene were added to chambers at final concentration of each 2 ppm. The samples were taken from chambers in duplicate and BTEX concentrations were analyzed using a gas chromatograph equipped with flame ionization detector (GC-FID Younglin series YL6100). Calibrations were performed at the BTEX concentrations of 0.2, 0.3, 0.4, 0.5, 1, 1.5 and 2 ppm. Samples were taken daily by a CAR/PDMS fiber (75 μm) and then immediately were analyzed using GC-FID. At the end of first experiment, the plants were removed and the pots with the soil alone were replaced in the chambers, which were sealed and they were exposed to a new dose of BTEX. For better plant removal, the pots were watered well, and then the plants extracted gently removing as little soil as possible. Finally, the experiment was performed with empty chamber to test the leakage.

### Statistical analysis

Means (n = 3) and standard errors (SE) were calculated. Daily BTEX values obtained were subjected to ANOVA analysis. Differences between variables were reported as statically significant where p value was less than 0.05.

## Results and discussion

**Figure 1:**
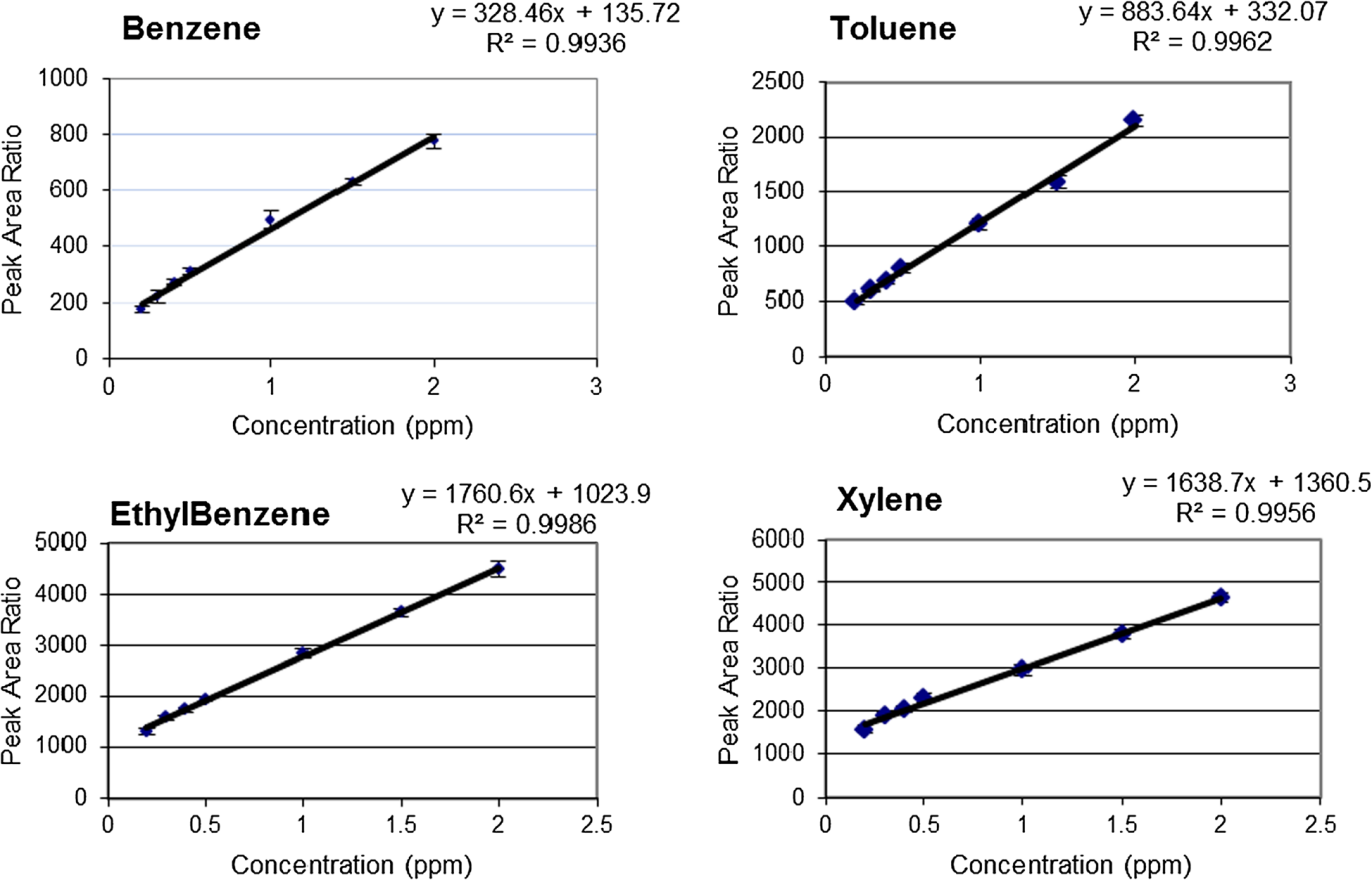
Calibration curves of benzene, toluene, ethylbenzene and xylene (n = 3).

**Figure 2:**
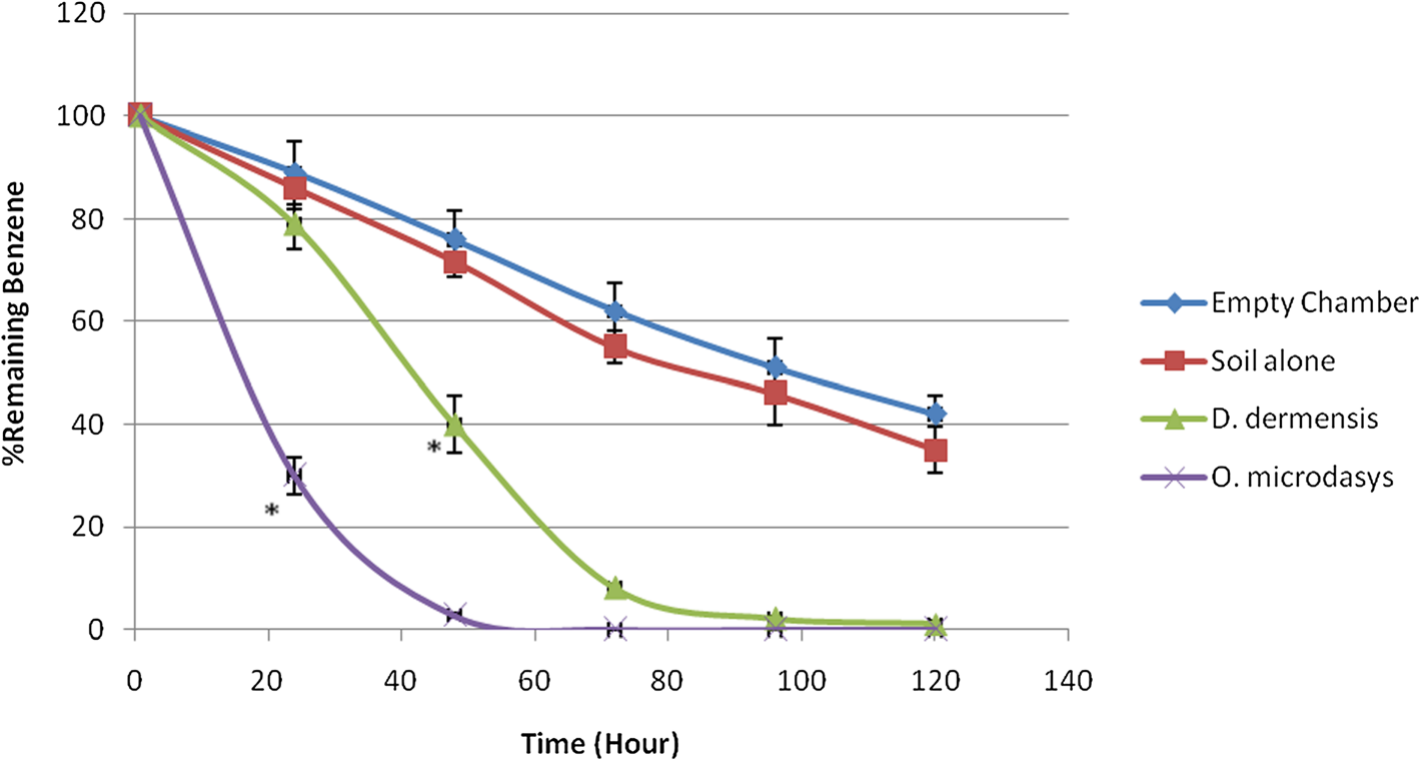
**Benzene removal capacity of *D. dermensis *and *O. microdasys*. **Benzene concentration was 2 ppm. Data are presented as means ± SEM, n = 3, * = p < 0.05.

**Figure 3:**
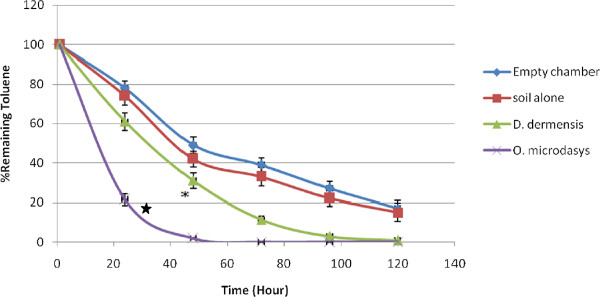
**Toluene removal capacity of *D. dermensis *and *O. microdasys*.** Toluene concentration was 2 ppm. Data are presented as means ± SEM, n = 3, * = p < 0.05.

**Figure 4:**
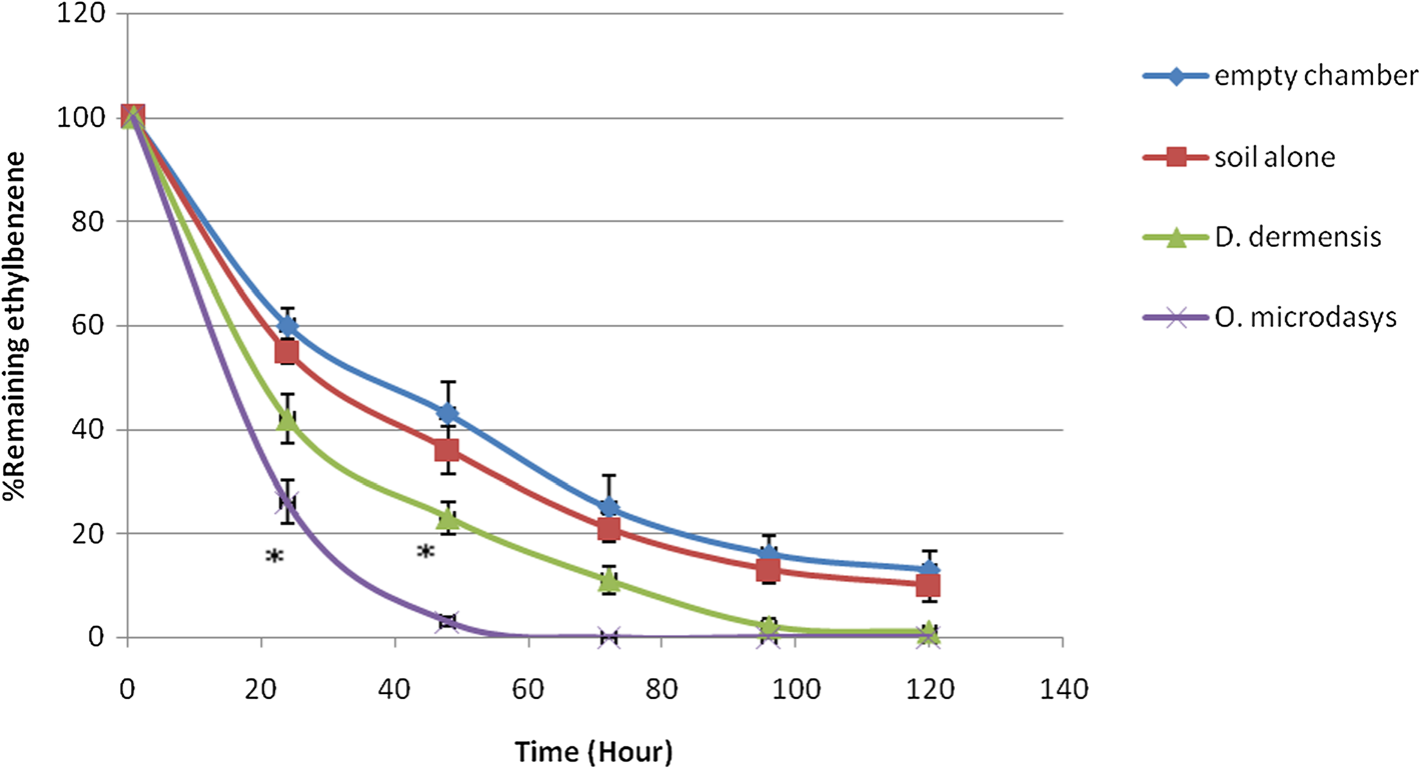
**Ethylbenzene removal capacity of *D. dermensis *and *O. microdasys*.** Ethylbenzene concentration was 2 ppm. Data are presented as means ± SEM, n = 3, * = p < 0.05.

**Figure 5:**
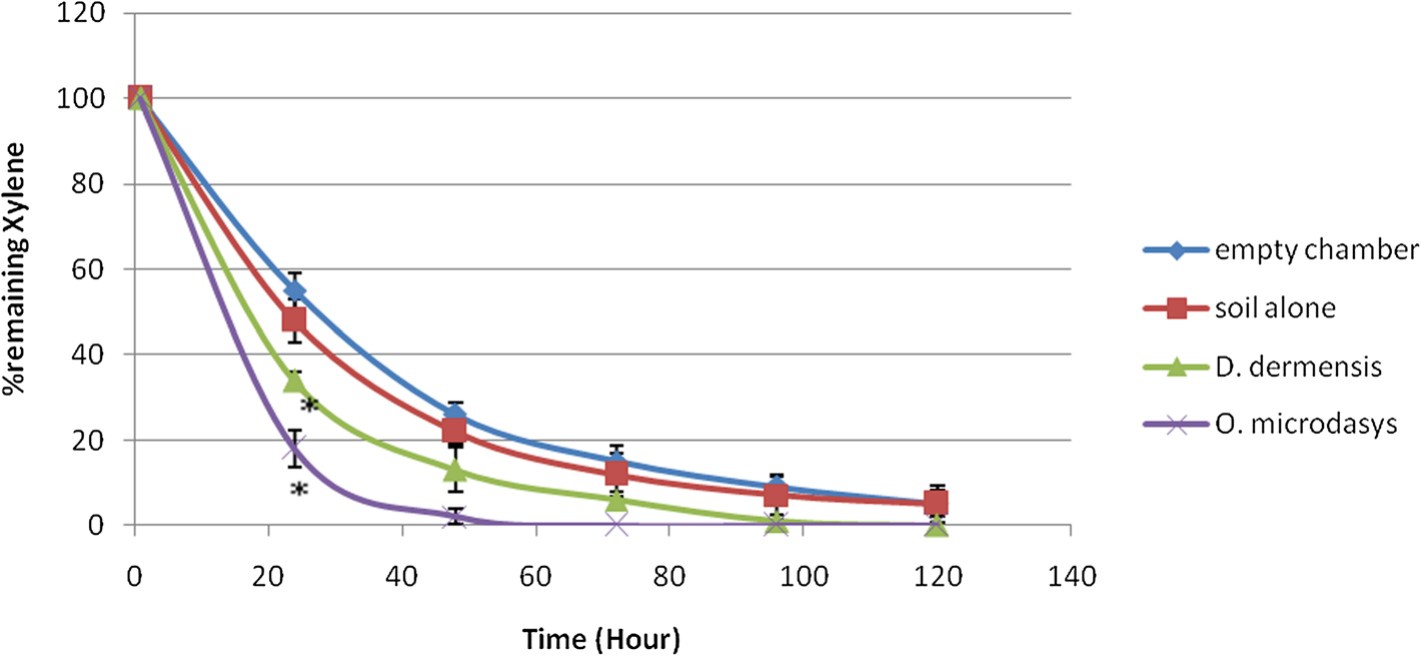
**Xylene removal capacity of *D. dermensis *and *O. microdasys*.** Xylene concentration was 2 ppm. Data are presented as means ± SEM, n = 3, * = p < 0.05.

Typical calibration curves of benzene, toluene, ethylbenzene and xylene (n = 3) in Tedlar bag air are shown in Figure 1. The BTEX concentrations of 0.2-2 ppm were used in these experiments to cover the range corresponding to the values established inside the chambers. Linear relationships between the concentrations of benzene, toluene, ethylbenzene and xyleneand peak area ratio were obtained (r^2^ = 0.9936, 0.9962, 0.9986 and 0.9956, respectively). BTEX concentrations could be decreased by by chemical destruction, absorption to the chamber and destruction by soil bacteria. To avoid the effects of these factors, the results of the plants were compared with the soil results and the differences between them were significant. Both *D. deremensis* and *O. microdasys* significantly reduced concentration of benzene, toluene, ethylbenzene and xylene in the chambers (Figures 2, 3, 4, 5).

Some of the plants have the capacity to remove air concentrations of VOCs, which knows as phytoremediation. Various plants have different capacities, which in this study the removal ability of the *D. dermensis* and *O. microdasys* plants were expressed based on leaf area, cleaned cubic meter of the air and both of them. Kim et al reported the ability of a few plants to remove volatile substances from indoor area [14].

*O. microdasys* was able to remove 2 ppm of benzene from air in the test chambers completely after 48 hours. However, *D. dermensis* could remove benzene from air in the test chambers completely after 105 hours. The removal rates of benzene in the test chambers were 3.2 and 1.46 mg/ m^3^d^1^ for *O. microdasys* and *D. dermensis*, respectively. Also, the removal rates of benzene from air in the test chambers were 1.18 and 0.52 mg/ m^2^d^1^ based on leaf area of *O. microdasys* and *D. dermensis*, respectively. On the other word, *O. microdasys* and *D. dermensis* abilities were 23.2and 10.6 mg/ m^3^m^2^d^1^, respectively. However, David Moerlein investigated the ability of S. *trifasciata*, F. *robusta*, C*. seifrizii* and D. *dermensis* in laboratory to reduce total air borne inorganic and organic particles 10 microns or smaller in diameter. The plants did not significantly affect total particles in rooms [26]. Corenjo et al studied the ability of eight species of plants to remove benzene from air. The best removal rate of benzene from air in chamber was 8.5 μg /g^1^d^1^ that removed by the P. *Domesticum* plant [27]. Their results confirmed the results of present project.

*O. microdasys* was able to remove 2 ppm of toluene from air in the test chambers completely after 55 hours. However, *D. dermensis* could remove toluene from air in the test chambers completely after 120 hours. The removal rates of toluene in test chambers were 1.47 and 0.67 mg/ m^3^d^1^ for *O. microdasys* and *D. dermensis*, respectively. Also, the removal rates of toluene from air in the test chambers were 0.54 and 0.24 mg/ m^2^d^1^ based on leaf area of *O. microdasys* and *D. dermensis*, respectively. On the other word, *O. microdasys* and *D. dermensis* abilities were 10.7 and 4.86 mg/ m^3^m^2^d^1^, respectively. Yang et al used 28 different plant species to determine their removal abilities. They showed that these plants could decrease toluene and benzene concentrations in a closed jar [28]. However, none of these plants had removal ability as much as O. microdasys and D. dermensis plants used in the present study.

Four different plant species were studied by Yoo et al to determine their removal abilities. Their removal abilities for benzene and toluene was good,which confirmed the results of present study [29]. However, their abilities were less than that of O. microdasys and D. dermensis plants.

The removal rates of benzene by the D. *Janet Craig*, E. *aureum*, D. *marginata*, S. *amate*, S. *petite,* S. *sensation* and H. *forsteriana* were studied by Orwell et al, and they were more than that of O. microdasys and D. dermensis plants [19].

O. microdasys plant can remove 2 ppm concentration xylene from air in test chambers completely after 47 hours. However, D. dermensis can remove xylene from air in test chambers completely after 98 hours. The removal rates of xylene in test chambers are 4.42 mg/ m^3^d^1^ and 2.12 mg/ m^3^d^1^ for O. microdasys and D. dermensis, respectively. Also, the removal rates of xylene from air in test chambers are 1.64 mg/ m^2^d^1^ and 0.76 mg/ m^2^d^1^ based on leaf area of O. microdasys and D. dermensis, respectively. On the other word, *O. microdasys* and *D. dermensis* abilities were 32.03 and 15.36 mg/ m^3^m^2^d^1^, respectively. Orwell et al reported the removal ability of S. *Sweet Chico* and D. *Janet Craig* plants for toluene and xylene. S. *Sweet Chico* and D. *Janet Craig* plants were prior to the plants used in the present study for removing toluene from air. However, the ability of O. *microdasys* for removing xylene from the air was prior to S. *Sweet Chico* and D. *Janet Craig* plants [30].

O. microdasys plant can remove 2 ppm concentration ethylbenzene from air in test chambers completely after 57 hours. However, D. dermensis can remove ethylbenzene from air in test chambers completely after 100 hours. The removal rates of ethylbenzene in test chambers are 3.65 mg/ m^3^d^1^ and 2.1 mg/ m^3^d^1^ for O. microdasys and D. dermensis, respectively. Also, the removal rates of ethylbenzene from air in test chambers are 1.35 mg/ m^2^d^1^ and 0.76 mg/ m^2^d^1^ based on leaf area of O. *microdasys* and D. *dermensis*, respectively. On the other word, *O. microdasys* and *D. dermensis* abilities were 26.45 and 15.22 mg/ m^3^m^2^d^1^, respectively.

In authors knowledge, there was not found any study work on the removal of the ethylbenzene so far.

## Conclusion

If an office containing 2.5 ppm of each of BTEX and had an approximate volume of 30 m^3^, it contains 16, 8, 22 and 22 mg/m^3^ benzene, toluene, xylene and ethylbenzene, respectively. Using ten of O. microdasys pots with the same size used in this study, can remove benzene, toluene, xylene and ethylbenzene totally after 36, 40, 30 and 39 hours, respectively.

The authors recommended studying the efficiency of the plants for removal of BTEX from air at higher range of concentrations such as 20-30 ppm.

In conclusion, there are a few studies reported the ability of different plant species in removing organic chemicals from the air. Various plants have different abilities for removal of air borne inorganic and organic particles. The best plant is one with higher removal rate, which could be recommended to be used indoor especially in high air polluted area.

## Abbreviations

VOCs: Volatile organic compounds; BTEX: Benzene, toluene, xylene and ethylbenzene; SPME: Solid phase microextraction

## Competing interests

The authors declare that they have no competing interests.

## Authors’ contributions

All authors precipitated in conception and design, generation of data, analysis of data, interpretation of data, drafting of manuscript, revision of manuscript and approval of the final draft.
